# Contribution of Extramedullary Hematopoiesis to Atherosclerosis. The Spleen as a Neglected Hub of Inflammatory Cells

**DOI:** 10.3389/fimmu.2020.586527

**Published:** 2020-10-26

**Authors:** Victoria Fernández-García, Silvia González-Ramos, Paloma Martín-Sanz, Antonio Castrillo, Lisardo Boscá

**Affiliations:** ^1^ Instituto de Investigaciones Biomédicas Alberto Sols (CSIC-UAM), Madrid, Spain; ^2^ Centro de Investigación Biomédica en Red en Enfermedades Cardiovasculares (CIBERCV), Madrid, Spain; ^3^ Centro de Investigación Biomédica en Red de Enfermedades Hepáticas y Digestivas (CIBERehd), Madrid, Spain; ^4^ Unidad de Biomedicina, (Unidad Asociada al CSIC), Instituto de Investigaciones Biomédicas Alberto Sols (CSIC-UAM) and Universidad de Las Palmas, Gran Canaria, Spain; ^5^ Instituto Universitario de Investigaciones Biomédicas y Sanitarias, Grupo de Investigación Medio Ambiente y Salud, Universidad de Las Palmas de Gran Canaria, Las Palmas, Spain

**Keywords:** hematopoiesis, atherogenesis, extramedullar manifestations, inflammation, spleen

## Abstract

Cardiovascular diseases (CVDs) incidence is becoming higher. This fact is promoted by metabolic disorders such as obesity, and aging. Atherosclerosis is the underlying cause of most of these pathologies. It is a chronic inflammatory disease that begins with the progressive accumulation of lipids and fibrotic materials in the blood-vessel wall, which leads to massive leukocyte recruitment. Rupture of the fibrous cap of the atherogenic cusps is responsible for tissue ischemic events, among them myocardial infarction. Extramedullary hematopoiesis (EMH), or blood cell production outside the bone marrow (BM), occurs when the normal production of these cells is impaired (chronic hematological and genetic disorders, leukemia, etc.) or is altered by metabolic disorders, such as hypercholesterolemia, or after myocardial infarction. Recent studies indicate that the main EMH tissues (spleen, liver, adipose and lymph nodes) complement the hematopoietic function of the BM, producing circulating inflammatory cells that infiltrate into the atheroma. Indeed, the spleen, which is a secondary lymphopoietic organ with high metabolic activity, contains a reservoir of myeloid progenitors and monocytes, constituting an important source of inflammatory cells to the atherosclerotic lesion. Furthermore, the spleen also plays an important role in lipid homeostasis and immune-cell selection. Interestingly, clinical evidence from splenectomized subjects shows that they are more susceptible to developing pathologies, such as dyslipidemia and atherosclerosis due to the loss of immune selection. Although CVDs represent the leading cause of death worldwide, the mechanisms involving the spleen-atherosclerosis-heart axis cross-talk remain poorly characterized.

## Introduction: Classical *vs.* Extramedullary Hematopoiesis

Hematopoiesis is the process by which blood cellular components are formed. It occurs from embryonic development to adulthood in order to produce and replenish the blood system. Hematopoietic stem and progenitor cells (HSPCs), with self-renewal and differentiation properties, can be employed as a model system to understand tissue stem cells´ fate and their role in aging, inflammation, atherogenesis and cancer (1–7).

Blood contains more than 10 different lineages: leukocytes represent specialized cells that participate in innate and adaptive immunity; erythrocytes are responsible for the transport of O_2_ and CO_2_, while megakaryocytes generate platelets for blood clotting and wound healing. In fact, blood is one of the most regenerative and plastic tissues (8–11). Every minute millions of senescent blood cells are eliminated and the replaced cells are submitted to ‘physiological’ scanning to avoid adverse actions. The life span of the different cell types varies from hours to years (3, 12, 13). The composition of blood cells in vertebrates involves three waves of hematopoiesis: the primitive, the transient and the definitive. The primitive step involves non-pluripotent erythroid progenitors without renewal capacity, and erythrocytes and macrophages originating from the yolk sac. The main role of this wave is to provide red blood cells (RBCs) to facilitate embryonic tissue oxygenation. The intermediate wave involves transient hematopoiesis and it takes place in the blood islands to produce erythroid-myeloid progenitors. Definitive hematopoiesis occurs later in development and at different times, depending on the species. It is associated with pluripotent HSPCs that can give rise to all blood adult lineages (14–16). The definitive HSPCs of vertebrates are generated in the aorta-gonad-mesonephros (AGM) region of the embryo, and primarily migrate to the fetal liver, and finally to the bone marrow (BM), which is the location of HSPCs in adults (3, 12). Recently, the placenta has been recognized as an additional site that participates in the transition from the AGM to the fetal liver.

The properties of HSPCs differ at each site, reflecting the existence of niches that support their expansion and/or differentiation. As an example of this, HSPCs present in fetal liver have mitotic activity, while the adult BM HSPCs are largely inactive (14, 17). In humans, hematopoiesis begins in the yolk sac and this function is transferred to the liver before finally settling in the BM and the thymus (3). HSPCs are reported to move and establish in the BM as well as in peripheral tissues through binding of CXCR4 to CXCL12 (18). In addition to this CXCR4/CXCL12 axis, other molecules and metabolites have been associated with the mobilization and nesting of HSPCs in other organs, such as CSF/Kit, CCL2/CCR2 and the sphingosine-1-phosphate (S1P) and its receptor (S1PR) among others (1, 2, 19). The specific roles of these different axes of EMH have been identified using animal models targeting for these molecules (2, 20) and it has been proposed that they may contribute to specific diseases, such as the splenic CCL2/CCR2-dependent EMH in supporting tumor growth (19).

### Anatomical Sites of EMH

The soft tissue outside the BM that produces blood cells is called extramedullary hematopoietic tissue and the process is defined as extramedullary hematopoiesis (EMH). It occurs when there is an insufficient or irregular production of blood cells from the BM (1, 18, 21–23). The most common triggers of EMH are certain chronic hematological disorders, such as chronic hemolytic anemias, thalassemias, myelofibrosis, atherogenesis, diffuse bone metastatic disease, lymphoma and leukemia (11, 24–30). Other pathological conditions, like infection or metabolic stress, can also promote EMH. Further research on the cellular and molecular mechanisms involved in EMH are important to prevent its pathological activation (31–33). Mobilized peripheral HSPCs are considered key players for inducing EMH (18).

The anatomical sites commonly involved in EMH include the spleen, liver, lymph nodes and paravertebral regions, although other organs and tissues may participate (heart, thymus, kidney, adrenal gland, prostate, pleura, skin, adipose tissue and nerves, among others) (18, 34, 35). There are several imaging studies of EMH, both in common and unusual anatomical locations (18, 21). Heterogeneous, soft-tissue masses poorly irrigated and usually interspersed with fat areas, display positive signals for EMH using computed tomography (22). Ultrasound techniques show that these areas are non-calcified and vascularized solid masses. Using magnetic resonance imaging, EMH appear as heterogeneous, lipid-loaded domains. Techniques such as technetium-99m colloidal imaging can be helpful in diagnosing EMH in a suspicious area by confirming the presence of BM components in it. Abdominal EMH usually appears as hepatosplenomegaly with or without focal soft tissue areas in the liver, spleen, peritoneum, and perirenal space (22). The ability of HSPCs to move from the BM to accommodate and function in extramedullary tissues is quite complex and is currently far from their complete understanding (19). This is an important issue, for example in oncologic patients requiring hematopoietic stem cell transplant to treat myeloproliferative neoplasm-associated myelofibrosis. These patients exhibit splenomegaly and an intense EMH (1, 36, 37).

### Establishment of HSPCs in Hematopoietic Sites

The establishment of HSPCs in hematopoietic locations is mediated by the expression of CXCL12 and vascular cell adhesion molecule-1 (VCAM-1), which bind to CXCR4 and to very late antigen-4 (VLA-4), respectively (29, 38–41). It is well known that, after infection, in both classical BM and EMH hematopoiesis, HSPCs are activated to produce mature lineage cells to fight pathogens. This process is of pathophysiological relevance since the immune cells have a short life and are eliminated during infection. Several studies confirm HSPCs activation not only after bacterial infection, but also in polymicrobial, viral, and fungal challenges. Immune cells are known to detect pathogen-associated molecular patterns (PAMPs) by their Toll-like receptors (TLRs), leading to an increased proinflammatory response. HSPCs that express TLRs can respond directly to infection or inflammation, and differentiate into specific cell lineages. This HSPCs activation also promotes the recruitment of these cells to EMH sites, where they are capable of generating neutrophils and monocytes. EMH can also be enhanced by a wide range of proinflammatory cytokines and other molecules that mediate these responses, such as IFN-γ, IFN-α, IL-6, IL-5, IL-1, G-CSF, and M-CSF (18). Moreover, psychosocial stress has been reported to increase blood cell progenitors production in the BM and its mobilization to the spleen, where they establish persistent myelopoiesis (33, 42). Therefore, increased production of monocytes and neutrophils occurs in a wide range of diseases, from anxiety to atherosclerosis (25–27, 33, 42–46). In fact, ectopic erythropoiesis in the spleen tends to improve anemia caused by stress (11, 33, 42). In addition, administration of isoprenaline promotes the mobilization of HSPCs to the spleen, which reveals a participation of the β-adrenergic pathways in this process and a crosstalk between the nervous and the immune systems (47, 48). It has been shown that extramedullary production of CD11b^+^ cells continues for at least 24 days after challenge (33). Conversely, therapeutic stimulation of the nicotinic acetylcholine receptor alpha 7 reduces splenic monocyte mobilization and antagonizes atherogenesis (49).

## Splenic Hematopoiesis

### The Spleen: Structure/Function Interplay and Its Impact on Leukocyte Action

The spleen, the body’s largest secondary immune organ, is a highly vascularized lymphopoietic tissue. Dysfunction or injury in the spleen causes significant loss of blood cells, either from the parenchyma or from arteries and veins that supply it (50–52). In human adults, spleen size is up to 250 g and *ca*. 13 cm long (21, 52). The spleen displays several essential functions: hematological (maturation of RBCs, efficient removal of abnormal cells by phagocytosis, iron recycling and removal of particles, such as opsonized microorganisms or cells coated with antibodies), immunological (humoral and cell-mediated immunity) and metabolic homeostasis (23, 53, 54). It is integrated in the regulation of local immune responses but also in systemic immunity, thus participating in the development of certain inflammatory and chronic disorders that will be discussed (51, 54–56). The spleen has two compartments that are very different in their architecture, vascular organization, morphology, cell composition and physiological functions: the red pulp (RP) and the white pulp (WP), separated by the marginal zone (MZ). The organ is surrounded by a capsule of dense fibrous tissue, elastic fibers and smooth muscle cells. The outermost layer of the splenic capsule is composed of mesothelial cells. Irregularly spaced trabeculae of smooth muscle and fibroblastic tissue emanate from the capsule in the splenic parenchyma and contain blood and lymphatic vessels, and nerves. Lymphatic vessels are efferent vessels through which lymphocytes migrate to splenic lymph nodes (57–60).

The RP constitutes a blood filter that removes foreign material, damaged erythrocytes (for iron recycling), and platelets. In rodents, it is a place of EMH (1, 23, 61). It is composed of a three-dimensional mesh of splenic cords and venous sinuses. The highly active RP macrophages are phagocytic cells that remove old and damaged RBCs and particles that flow through the blood (51, 55, 62). EMH is common in the RP of rodents, especially in fetal and neonatal animals (1).

The spleen contains almost a quarter of the body’s lymphocytes and initiates immune responses to blood antigens, especially in the WP, which surrounds the central arterioles and is subdivided into the periarteriolar lymphoid sheath (PALS), the follicles and the MZ (15, 58). It consists of lymphocytes, macrophages, dendritic cells (DCs), plasma cells, arterioles and capillaries in a reticular framework similar to that found in the RP. The PALS is composed of lymphocytes and concentric layers of reticular fibers and flattened reticular cells (63). The internal PALS cells are mainly CD4^+^ T cells, although small amounts of CD8^+^ T cells are also present, as well as interdigital DCs and migrating B cells. The external PALS is formed by B and T lymphocytes, macrophages and, after antigenic stimulation, newly formed plasma cells. The follicles are continuous in the PALS and are mainly composed of B cells with a smaller number of follicular DCs and CD4^+^ T cells, but usually do not contain CD8^+^ T cells. They contain germinal centers, which are formed after antigenic stimulation and contain macrophages and apoptotic B cells.

The MZ of the spleen is composed of a wide variety of cell types, some of which have a fixed position therein, such as macrophages in the MZ, metallophilic macrophages on the edge and, to a lesser extent, B cells in the MZ (52, 58, 59, 64). T lymphocytes, small B cells and DCs, reside only temporarily in the MZ. Therefore, continuous flow of blood-borne immunocompetent cells together with the sessile cell populations, make the MZ a dynamic area suited for antigens recognition and processing. There is no other lymphoid organ in which such a unique combination of cells and functions can be found. Metallophilic macrophages of the MZ and the marginal sinus separate the MZ from the PALS and the follicles. Metallophilic macrophages in the MZ are a single subset of macrophages in the inner border of the MZ adjacent to the PALS and follicles (58, 65). They can be visualized by staining with silver and with the MOMA-1 monoclonal antibody (that recognizes CD169, also known as Siglec-1). The vessels that feed the PALS capillary bed and follicles are delineated by MADCAM1^+^, the sinus-coated endothelial cells. Peripheral to the marginal sinus, is the thick outer ring of the MZ, with reticular fibroblasts, macrophages, DCs and B cells. Macrophages of the MZ are another population of splenic macrophages that are selectively characterized by the expression of scavenger receptor MARCO and by SIGN-R1 lectin (a member related to the DC-SIGN family and positive for ERTR-9 monoclonal antibody) (59, 64, 66–69). Although not all the potential functions of the metallophilic macrophages of the MZ are known, they are important for the removal of foreign particles, bacteria and viruses. They express a series of pattern recognition receptors (PRRs) such as TLRs and CD169 that bind sialic acid motifs present in microbes. These receptors are essential to detect pathogens and to lead to efficient phagocytosis. The antigenic fragments that are produced can be absorbed by DCs, which enter the spleen through the blood as part of a mobile immune surveillance system and present them to T cells, promoting the clustering and enrichment of antigen-specific T cells. Antigens in the MZ can also be directly associated with memory B cells that migrate into the PALS and present the antigen to T cells. Therefore, the MZ also acts as a lymphocyte trafficking site (52, 58).

### Splenic Extramedullary Hematopoiesis and Atherosclerosis: The Missing Links

**Figure 1:**
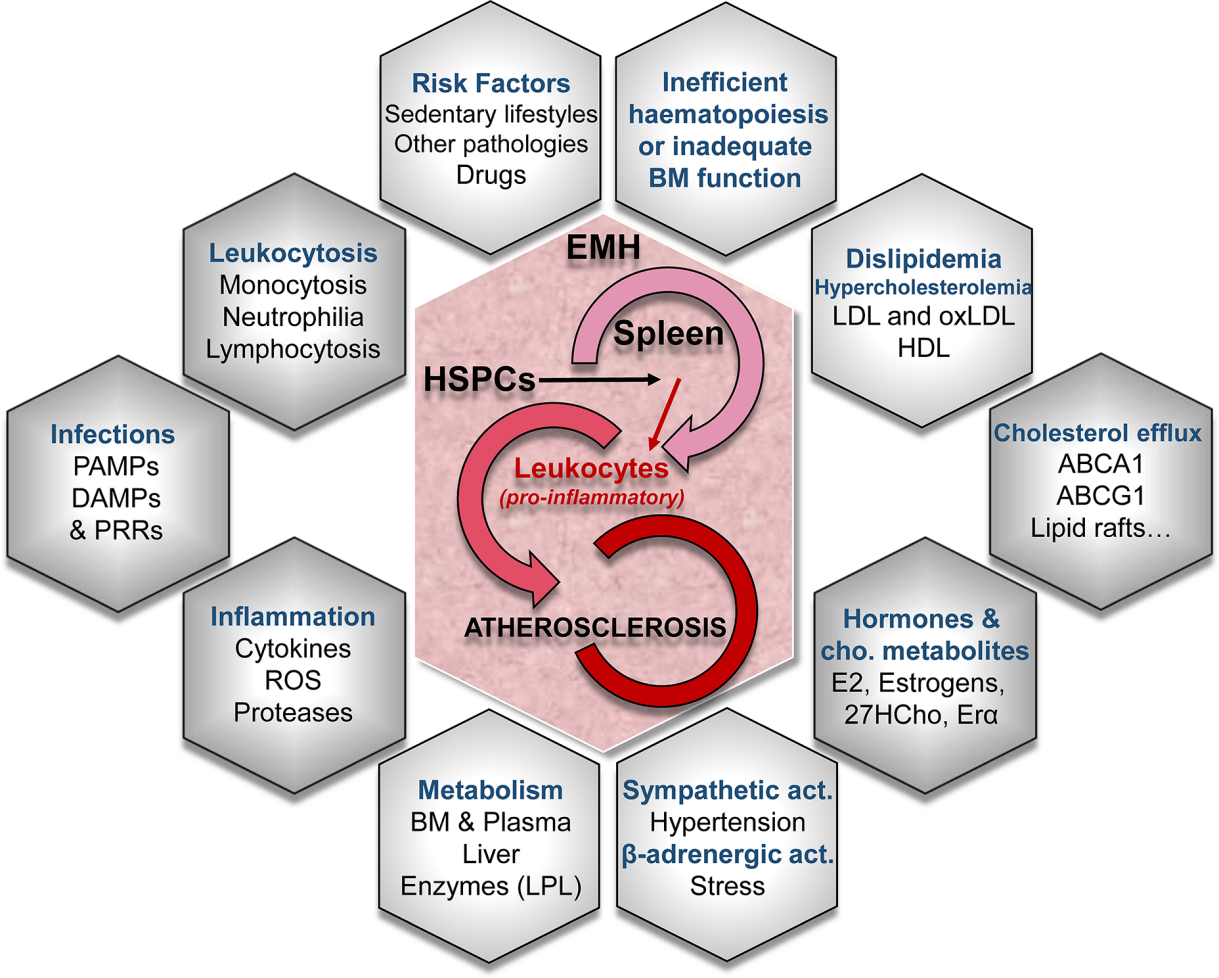
Conditioning factors in extramedullary hematopoiesis (EMH) -especially in the spleen- leading to atherosclerotic disease. Different mechanisms lead to EMH, the generation of new leukocyte progenitors and their role in the progression of the atherosclerotic disease. EMH is caused by truncated normal hematopoiesis or inadequate bone marrow (BM) function. Several risk factors, the activation of the sympathetic nervous system and/or the β-adrenergic activation are involved in the turn-on of EMH mechanisms. The concurrence of EMH with alterations in lipid metabolism and cholesterol-efflux pathways synergize in the aggravation of the proatherogenic pathways. LDL, low-density lipoprotein; oxLDL, oxidized low-density lipoprotein; HDL, high-density lipoprotein; ABCA1 and ABCG1, cholesterol cassette transporters; E2, 17β-estradiol; 27HC, 27-hydroxycholesterol; ERα, estrogen receptor α; LPL, lipoprotein lipase; ROS, reactive oxygen species; PAMPs, pathogen-associated molecular patterns; DAMPs, damage-associated molecular patterns; PRRs, pattern recognition receptors.

Cardiovascular diseases (CVDs) are the main cause of mortality worldwide, being atherosclerosis one of the underlying agents of these pathologies. The deposition of cholesterol-rich lipoproteins in the arterial wall initiates the atherosclerotic process, triggering leukocyte recruitment and chronic inflammation. Aforetime, atherosclerosis was thought to be only the consequence of the progressive lipid accumulation in the blood vessels. The current view is that it is a much more complex process involving both lipids and immune cells **(** **Figure 1** **)** (29, 70–72). Recently, a strong association between leukocytosis and CVDs has been demonstrated, thus highlighting innate immunity as a hallmark in the onset and progression of these pathologies (29, 71, 73). While inflammation associated with atherosclerosis may contribute to this relationship, there is also evidence that leukocytosis directly increases atherosclerosis and thrombosis (26, 27, 70, 74, 75). The numerous risk factors for CVDs, including obesity, smoking, sedentary lifestyles and metabolic syndrome (which in turn includes individual components, such as dyslipemia and low HDL), are associated with leukocytosis (42, 76).

HSPCs can produce monocytes outside the BM (18, 22). In addition, the mechanisms of proliferation and differentiation of HSPCs remain partly unknown, but epigenetic modifications on the HSPCs appear to be important in the memory of trained innate immunity (77, 78). Several findings indicate that extramedullary anatomical sites complement the hematopoietic function of the BM producing circulating inflammatory cells that infiltrate atherosclerotic lesions (2, 72, 74, 79, 80). Preclinical studies of atherosclerosis in mice determined that the spleen contains a reservoir of monocytes, which further yield Ly-6C^high^ monocytes to the growing atheroma layer. These monocytes express pro-inflammatory interleukins (such as pro-IL1) and have proteolytic capacity, contributing to the remodeling of the atheroma and favoring plaque instability. In addition to this, they exhibit increased production of reactive oxygen species, promoting the oxidation of LDL, which results in macrophages loaded with lipids (foam cells) (55, 80). Regarding the ability of HSPCs to produce different cell lineages, it has been shown the existence of specific signals that rule the selection processes, but also memory epigenetic modifications that accelerate and condition innate immune function responses (29, 45, 77, 78, 81). Interestingly, several studies demonstrate that HSPCs, not only from BM but also cells produced *via* EMH (73, 74, 80, 82, 83) are associated with CVD (i.e., coronary heart disease and stenosis) and give rise to inflammatory cells (82).

### Contribution of Splenic Neutrophilia and Monocytosis to Atherosclerosis

Neutrophilia and monocytosis have been specifically associated with CVDs and with atherosclerotic plaque burden in both prospective and cross-sectional studies. In animal models, monocytosis has been associated to atherosclerosis (6, 29, 42, 44, 45, 72, 73, 84). More than 30 years ago a relationship between dietary hypercholesterolemia, monocytosis and atherosclerosis was observed in pig and rabbit models (85, 86). In hypercholesterolemic pigs, an increased colony forming units in the BM was observed, which was also corroborated lately in other experimental models (45). Increased HSPCs elevate monocyte production in the BM and other extramedullary tissues, resulting in the accumulation of cells that worsen the atherosclerotic disease due to lipid accumulation. Additional work has highlighted that the disturbance of cholesterol pathways may set this HSPC-dependent monocytosis (29, 44, 82, 87). Furthermore, differential macrophage polarization (55, 84, 88) (proinflammatory or M1; anti-inflammatory/pro-resolution or M2a/M2b), could drive the outcome of the inflammation and the pathology (89–92). Dietary hypercholesterolemia in the *Apoe* ^−/−^ mouse model of atherosclerosis is associated with progressive monocytosis and an increase in the subgroup of Ly-6C^hi^ monocytes (CCR2^+^), which is more common in lesions than Ly-6C^lo^ monocytes. Ly-6C^hi^ monocyte subset is thought to differentiate into an inflammatory phenotype (55, 80). In advanced atheromata, in contrast to previous views on macrophage proliferation, macrophages can proliferate inside the lesions, through an Scavenger Receptor Class A (SRA)-dependent pathway (71, 93, 94). Cytokines related to leukopoiesis and monocyte mobilization from BM, like M-CSF, G-CSF, and MCP-1, are produced in an enhanced manner by macrophages carrying *Abca1*/*Abcg1* deletion, supporting the role of the dyslipemia in these processes (25, 27, 44, 95). Mouse models that were deficient in these genes had hematopoietic progenitors and/or monocyte-derived cells more prone to accumulate cholesterol and consequently, to transport cholesterol to atherosclerotic lesions (83, 95, 96). Furthermore, cholesterol homeostasis appears to support hematopoietic stillness and quiescence of HSPCs. These cells show enhanced expression of *Abca1*, *Abcg1* and *Apoe*, all of them essential cholesterol-efflux genes. Mice with defects in cholesterol flow pathways (deficiencies of ABCA1 and ABCG1 cassette transporters) show a dramatic increase in HSPCs and EMH (25, 27, 32, 83, 96). This favors the tendency of hematopoietic lineages’ differentiation towards granulocytes, instead to macrophages in the BM, leading to the deterioration of osteoblasts and to the decrease in the production of CXCL12 (SDF-1) by mesenchymal progenitors. Hypercholesterolemic primed HSPCs have been suggested to lead to atherogenic macrophages that release higher amounts of TNF-*α*, IL-6, and MCP-1. These hypercholesterolemic-primed progenitors also produce myeloid cells that enter the atheroma and increase the lesion size (79, 84). Not only is the quantity of circulating monocytes important to these processes, but also the physiological sites of generation as well, because spleen-produced monocytes appear to have a pro-atherogenic phenotype (29, 83).

### Hypercholesterolemia and Splenic EMH in Atherosclerosis

Not only has systemic hypercholesterolemia been associated with monocytosis, but also with severe neutrophilia as well (27, 97). Current evidence also suggests that neutrophilia may promote early lesion development. Monocytosis in *Apoe* ^−/−^ mice suggests a simultaneous increase in production and a decrease in cell clearance; with the underlying mechanism due in part to an increase in granulocyte colony stimulating factor (G-CSF) coupled with cholesterol dysregulation which increases HSPC proliferation and skewing of leukocyte production towards monocytes and neutrophils (5, 44, 45, 97, 98). In addition to this, cholesterol-related pathways control the proliferation of progenitor hematopoietic stem cells (25, 81, 82, 99). Therefore, there is a particular link between metabolism and inflammation whose study could contribute to the development of different strategies for the treatment of CVDs (79). Monocytes circulate in the blood and patrol the vascular endothelium. Under inflammatory conditions, they accumulate at the target sites and mature to macrophages or DCs. In the arteries, they differentiate into macrophages, which accumulate oxidized lipoproteins in the atheroma layer and give rise to foam cells, contributing to the necrotic nucleus of the lesions. Although monocytes are thought to arise exclusively in the BM, HSPCs easily move from their niches in the BM, accumulating in the periphery where they differentiate. This EMH phenomenon gives rise to erythrocytes, platelets, granulocytes and DCs, but its regulation is a controversial issue (4, 55, 100).

### Hypercholesterolemia and Splenic Proliferation of HSPCs

The spleen also contains proliferating myeloid cell progenitor cells that give rise to its progeny *in vivo*. Using murine models of atherosclerosis and fate-mapping approaches, progenitor and hematopoietic cells have been shown to progressively move from BM to splenic RP. In the presence of GM-CSF and IL-3, cells expand clonally and differentiate into Ly-6C^high^ monocytes (7, 80). In addition, plasma lipids are closely related to monocytosis, exhibiting a direct correlation with total cholesterol and an inverse correlation with high-density lipoprotein (HDL) in plasma (26, 45, 97). Recently, the role of HDL in promoting the outflow from cholesterol-loaded foam cells, has been identified as a better marker of prognostic atherosclerosis than others (101). Reconstituted HDL (rHDL) infusions or ApoA-I transgenic models protected the athero-prone mice from the disease by preventing the formation of cholesterol-loaded cells, the adhesion of inflammatory cells and the activation of the endothelium (25, 75). Moreover, the scavenger receptor type BI (SR-BI), which is a high density lipoprotein (HDL) receptor, is expressed on HSPCs and is necessary for the antiproliferative effects of HDL on HSPCs (32, 44, 75, 96). However, there are contrasting studies that show that HDL facilitate the removal of cholesterol from cells, which in turn regulates HSPCs and the ontogeny of leukocytes, especially monocytes (44, 75). Deletion of the cholesterol efflux genes also resulted in an increase in monocytosis and neutrophilia, leading to an exacerbated progression of atherosclerotic lesions. Moreover, it also promoted leukocyte infiltration in other tissues (spleen, liver and intestine) and enhanced EMH. When HSPCs reach EM sites like the spleen, this immune secondary organ becomes an active hematopoietic site; hence, it constitutes a huge provider of myeloid cells that might eventually interact with the atheroma lesion. Thus, there is a connection between hypercholesterolemia, impaired cholesterol-flow pathways, monocytosis and atherosclerotic disease. Greater mobilization of HSPCs and EMH was reversed by increasing HDL levels in *Abca1* ^−/−^, *Abcg1* ^−/−^ and *Apoe* ^−/−^ mice or in a mouse model of myeloproliferative neoplasia mediated by the Flt3-ITD mutation. These results identify an emerging role of cholesterol-efflux in the control of HSPCs (32, 45). Beyond lipids and cholesterol impact on EMH and atherosclerosis, the influence of lipid rafts on hematopoiesis homeostasis and CVD has been studied. Highly ordered cholesterol and sphingolipid-rich regions of the plasma membrane (lipid rafts), contain receptors involved in HSPCs hematopoiesis (*e.g*. TGF-β1, GM-CSF, and IL-3 receptors). Many mechanisms modulate the distribution and composition of lipid rafts, but efficient cholesterol efflux is the most important one and, consistently, cholesterol pathways contribute to hematopoiesis homeostasis (88). Changes in the cholesterol-efflux in the cell membrane, as can happen in various pathologies, augment lipid raft content, leading to distend receptor occupancy and increasing downstream signaling. Stimulated lipid rafts contribute to protein dimerization, phosphorylation, or crosslinking, activating intracellular signaling pathways. Furthermore, in certain diseases, such as diabetes and obesity, exogenous fatty acid production causes an increase in lipid rafts and in inflammation (46).

### Role of ABC Transporters and Cholesterol Efflux on Splenic Myeloid Proliferation

ABC transporters are found in lipid rafts and their deficiency improves the formation of lipid rafts in HSPCs and myeloproliferation (32, 36). In addition to the well-known ABCA1, ABCG1, and ABCG4 transporters, other ABC transporters, such as AIBP (non-cellular autonomously acting protein binding protein apoA-I), have emerged as novel and relevant secreted proteins that regulate cholesterol-flow in hematopoiesis and they are potential new therapeutic targets. AIBP binds apoA-I and HDL, and therefore, increases cholesterol outflow, it disrupts lipid rafts in macrophages and DCs, and attenuates hyperlipidemia and atherosclerosis (102, 103). It can also bind to activated TLR4 in lipid rafts, recruiting HDL/ApoA-I, removing cholesterol from lipid rafts and dissociating the active TLR4 dimer. This mechanism reduces inflammatory signaling downstream of TLR4 (46). Oxysterols, molecules derived from oxygenated cholesterol, participate in the synthesis of bile acids and are important mediators in hematopoietic and immune pathways (99). Interestingly, hormones like 17β-estradiol promote HSPCs division and its receptor, estrogen receptor α (ERα) triggers sexual dimorphism of the hematopoietic stem cells division rate (81, 99). HSPCs express ERα and the binding of this receptor to estrogen promotes the self-renewal and proliferation of CD150^+^CD48^-^LSK cells, which further induce EMH. This situation is common during pregnancy, in which cholesterol plasma levels are increased (99). 27-hydroxycholesterol, another cholesterol metabolite, in combination of ERα, triggers the transposition of HSPCs from the BM and enhances EMH, thus being able to initiate or worsen atherosclerosis. During pregnancy, HSPCs proliferation and EMH are induced in order to maintain the rapid increase in the maternal blood volume (81, 104).

### Splenectomy, Splenomegaly, and Hypersplenism in CVDs

**Figure 2:**
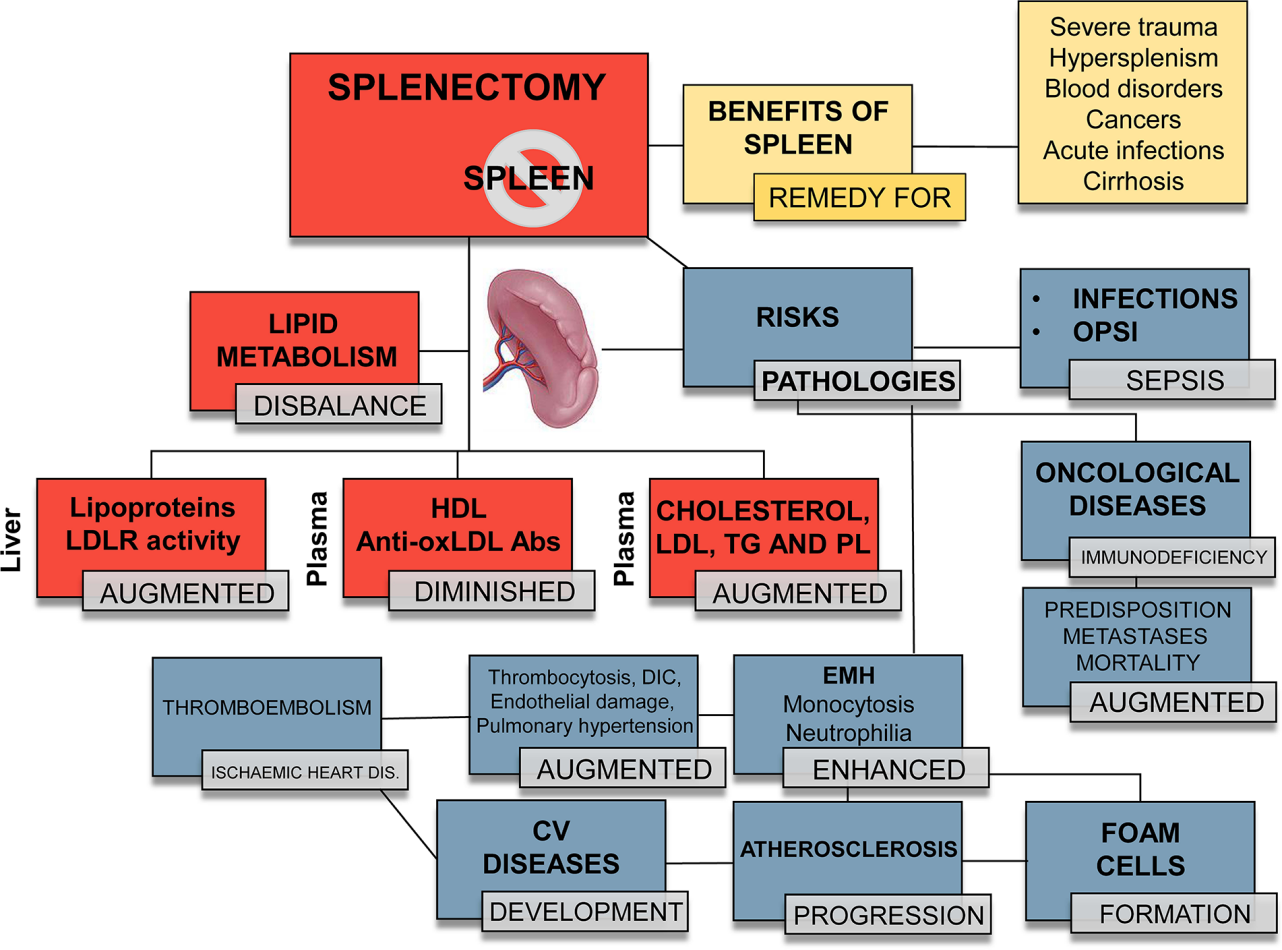
Different outcomes in splenectomized patients. *Orange boxes*: benefits of a fully functional spleen. *Red boxes*: summary of the changes that splenectomy exerts on the lipid profile and metabolism. Therapeutic/traumatic (total or partial) splenectomy provokes or aggravates specific pathophysiological processes. *Blue boxes*: risks associated to splenectomy. Overall, splenectomy leads to an increased susceptibility to infectious processes, with OPSI (overwhelming postsplenectomy infection) and sepsis being the fatal consequences. Increased EMH leads to an enhanced number of circulating leukocytes, mainly monocytes and neutrophils, which migrate to the atheroma plaque and trigger atherogenesis. EMH contributes to an increase in platelets, disseminated intravascular coagulation (DIC), thrombi, endothelial damage and hypertension, favoring the development of cardiovascular (CV) diseases, such as thromboembolism and ischemic heart disease. LDL, low-density lipoprotein; LDLR, low-density lipoprotein receptor; HDL, high-density lipoprotein; anti-oxLDL, antibodies against oxidized LDL; TG, triglycerides; PL, phospholipids.

The spleen plays an important role in lipid metabolism: elevated LDL, altered lipid values, and atherosclerosis are common consequences of surgical removal of the organ in animal models and in humans (105–108). These values ​​become normal when a splenic transplant is performed (109). Conversely, partial splenectomy and conservative procedures reduce these changes in lipid metabolism (105). Therefore, it is proposed that the spleen is an essential regulator of lipid metabolism and the immune-cell function (**Figure 2**) and this gives rise to propose what is called `the splenic factor´ (29, 105). Different studies support this view: higher cholesterol, triglycerides and phospholipid levels, and diminished HDL have been reported after splenectomy in rabbits and other animals; however, some researchers reported conflicting results (105, 110). In humans, splenectomy was related to increased LDL levels and alterations in serological lipid profiles (105, 107, 111, 112). Moreover, individuals with hypersplenism and splenomegaly (i.e.; Tangier disease patients) displayed hypocholesterolemia (30, 113–115), associated to an elevated activity of the mononuclear phagocytic system in diseases, such as malaria (116). However, as there are contradictory conclusions in these studies (117), the contribution of the spleen to the pathophysiology of inflammation and atherogenesis remains an open issue. This is due to the fact that the synthesis of lipoproteins and their excretion occurs mainly in the liver, and LDLR activity is modulated by many factors, including circulating PCSK9 levels (118, 119). Furthermore, splenectomy inhibits the pro-inflammatory activity of Kupffer cells in the liver, which are responsible of bacterial and lipoproteins clearance (120, 121). Moreover, neutralizing the proinflammatory activity of Th1 cells by activation of spleen memory B cells, as well as by the splenic reservoir of T*reg* cells, has been shown in several pro-atherogenic animal models to be effective against atherosclerosis (54, 122–127). Finally, the spleen is an essential immune tissue that filters the blood and responds to non-self-antigens and to oxLDL by generating anti-oxLDL antibodies, conferring protection against atherosclerosis (128–130). Indeed, immunization of *ApoE* ^−/−^ mice with anti-oxLDL antibodies has been shown to protect them against atherogenic challenges (131).

There are other connections between spleen and atherosclerosis, related to hematopoietic homeostasis. Hypertension-derived-mechanisms driven by the sympathetic nervous system regulate hematopoiesis and aggravate atherosclerosis and CVDs (43). Sympathetic activation in *Apoe* ^−/−^ mice compared to athero-prone and hypertensive mice models (BHP/*Apoe* ^−/−^) led to the degradation of the BM niche and the subsequent mobilization of HSPCs to other tissues, such as the spleen. In addition, this nervous system activation induces the release of proteases by neutrophils which cleave the CXCR4 receptor on HSPCs (43, 74). Moreover, myocardial infarction induces EMH and, in turn, this myelopoiesis increases the severity of the atherosclerotic disease (18, 55, 72, 73, 132).

### Pros and Cons of Splenectomy: Clinical Impact in Atherogenesis and CVDs

Circulating HSPCs significantly contribute to inflammatory diseases (4, 19, 26, 32, 74, 82). Accumulation of leukocytes in the arterial wall is one of the main characteristics of the chronic atherosclerosis. Lymphocytes, monocytes and neutrophils are essential for the development and progression of the disease. This change in the hematopoietic topographic hierarchy during inflammation entails important biological, diagnostic and therapeutic implications. Monocytes from extramedullary tissues, such as the spleen, belong to the inflammatory subset (Ly-6C^high^) and express proinflammatory factors, including reactive oxygen species and proteases (80, 133, 134). They accumulate in developing lesions, favoring an enhanced lipid intake and hypercholesterolemia that lead to atheroma progression. The spleen contribution to the disease has received some attention due to its clinical, social and health implications. The organ may be expendable, but this generally has important consequences, as explained in **Figure 2**. Splenectomy increases the risk of infection, with overwhelming post-splenectomy infection (OPSI) being one of the most worrisome complications. (135–138). OPSI consists of fulminant sepsis caused by encapsulated bacteria and can occur even long after surgery. Splenectomy also increases the risk of ischemic heart disease (136, 138, 139). During the past decades, splenectomy has been widely used as a remedy for those patients suffering spleen injuries or trauma (105, 140). Several studies attempted to clarify the link between higher mortality rates, infections, and other fatal conditions (such as pneumonia or myocardial ischemia) and trauma-induced splenectomies (105, 117, 128, 140, 141). These studies show that asplenia or partial splenic excision decreases the supply of immune cells, the elimination of particulate antigens and humoral immunity, and thus increasing the risk of infections (141–144). Additionally, the elevated platelet counts results in thromboembolism-prone subjects with a hypercoagulable condition and an increased risk of ischemic heart disease and mortality, again indicating another link between the spleen and the heart. In line with these abnormalities, reactive thrombocytosis has been shown to contribute to disseminated intravascular coagulation, endothelial damage, and pulmonary hypertension (105); however, there other studies that consider the benefits of splenectomy after experimental stroke (110).

## General Discussion

Nowadays, even if clinicians actively try to avoid splenectomy, there are several cases in which this surgical procedure is considered:

- Spleen rupture or severe trauma.
- Hypersplenism.
- Blood disorders (hemolytic anemia, idiopathic thrombocytopenic purpura, polycythemia vera, thalassemia, hereditary spherocytosis, sickle cell anemia Tangier disease, etc.).
- Acute infections or a large accumulation of pus with abscess in the spleen.
- Thrombosis in the blood vessels of the spleen or cirrhosis in the liver.
- Certain types of cancers (*e.g.* chronic lymphocytic leukemia, Hodgkin lymphoma, non-Hodgkin lymphoma, myeloproliferative dysplasia and hairy cell leukemia).
- Non-cancerous cysts or tumors inside the spleen when they enlarge or are difficult to remove completely.

Moreover, splenectomy is only performed after the failure of other therapeutic treatments (139, 145). Vascular occlusions, splenorrhaphies and partial splenectomies are some examples of current spleen-preserving procedures (105).

## Conclusions

Beyond the classic studies of cardiovascular risk factors and atherosclerosis disease, the spleen has been discovered as a key regulator of lipid metabolism and as an important source of inflammatory leukocytes for the progression of the atheroma layer. However, its complexity and intricate regulation are far from our complete understanding. Unraveling the interplay between metabolism (mainly cholesterol fluxes) and EMH appears to be a key issue in the development of new therapeutic strategies against atherosclerosis by focusing on specific targets.

## Author Contributions

VF-G wrote the paper, designed the figures and revised the manuscript. SG-R, PM-S, and AC provided intellectual input. SG-R also revised the manuscript. LB provided funding and intellectual input and discussed the information. The authors thank Mr Adrián Povo-Retana for the review and critical comments on the manuscript. All authors contributed to the article and approved the submitted version.

## Funding

The review was written by authors that work in a laboratory where projects are supported by: Ministerio de Economía, Industria y Competitividad, Ministerio de Ciencia, Investigación y Universidades, and Agencia Estatal de Investigación (SAF2017-82436-R, RTC2017-6283-1, PID2019-104284RB-I00/AEI/10.13039/501100011033), Centro de Investigación Biomédica en Red en Enfermedades Cardiovasculares (CB16/11/00222), Fundación Ramón Areces (CIVP18A3864), Consorcio de Investigación en Red de la Comunidad de Madrid, S2017/BMD-3686 and Fondo Europeo de Desarrollo Regional.

## Conflict of Interest

The authors declare that the research was conducted in the absence of any commercial or financial relationships that could be construed as a potential conflict of interest.
